# Optimization of irradiation dose to *Aedes aegypti* and *Ae*. *albopictus* in a sterile insect technique program

**DOI:** 10.1371/journal.pone.0212520

**Published:** 2019-02-19

**Authors:** J. Guillermo Bond, Adriana R. Osorio, Nancy Avila, Yeudiel Gómez-Simuta, Carlos F. Marina, Ildefonso Fernández-Salas, Pablo Liedo, Ariane Dor, Danilo O. Carvalho, Kostas Bourtzis, Trevor Williams

**Affiliations:** 1 Centro Regional de Investigación en Salud Pública (CRISP-INSP), Tapachula, Chiapas, Mexico; 2 Programa Moscas de la Fruta (SAGARPA-IICA), Camino a Cacaotales S/N, Metapa de Domínguez, Chiapas, Mexico; 3 Facultad de Ciencias Biológicas, Universidad Autónoma de Nuevo León, Nuevo León, Mexico; 4 El Colegio de la Frontera Sur, Tapachula, Chiapas, Mexico; 5 Insect Pest Control Laboratory, Joint FAO/IAEA Programme of Nuclear Techniques in Food and Agriculture, IAEA Laboratories, Seibersdorf, Austria; 6 Instituto de Ecología AC (INECOL), Xalapa, Veracruz, Mexico; University of Maryland, UNITED STATES

## Abstract

The sterile insect technique (SIT) may offer a means to control the transmission of mosquito borne diseases. SIT involves the release of male insects that have been sterilized by exposure to ionizing radiation. We determined the effects of different doses of radiation on the survival and reproductive capacity of local strains of *Aedes aegypti* and *Ae*. *albopictus* in southern Mexico. The survival of irradiated pupae was invariably greater than 90% and did not differ significantly in either sex for either species. Irradiation had no significant adverse effects on the flight ability (capacity to fly out of a test device) of male mosquitoes, which consistently exceeded 91% in *Ae*. *aegypti* and 96% in *Ae*. *albopictus*. The average number of eggs laid per female was significantly reduced in *Ae*. *aegypti* at doses of 15 and 30 Gy and no eggs were laid by females that had been exposed to 50 Gy. Similarly, in *Ae*. *albopictus*, egg production was reduced at doses of 15 and 25 Gy and was eliminated at 35 Gy. In *Ae*. *aegypti*, fertility in males was eliminated at 70 Gy and was eliminated at 30 Gy in females, whereas in *Ae*. *albopictus*, the fertility of males that mated with untreated females was almost zero (0.1%) in the 50 Gy treatment and female fertility was eliminated at 35 Gy. Irradiation treatments resulted in reduced ovary length and fewer follicles in both species. The adult median survival time of both species was reduced by irradiation in a dose-dependent manner. However, sterilizing doses of 35 Gy and 50 Gy resulted in little reduction in survival times of males of *Ae*. *albopictus* and *Ae*. *aegypti*, respectively, indicating that these doses should be suitable for future evaluations of SIT-based control of these species. The results of the present study will be applied to studies of male sexual competitiveness and to stepwise evaluations of the sterile insect technique for population suppression of these vectors in Mexico.

## Introduction

The mosquitoes *Aedes aegypti* and *Ae*. *albopictus* are the principal vectors of several emerging and re-emerging arboviruses of major importance in public health in tropical and subtropical regions worldwide [1]. Dengue (DENV), chikungunya (CHKV), yellow fever (YFV) and Zika virus (ZIKV) are mainly transmitted by *Ae*. *aegypti*, although *Ae*. *albopictus* is playing an increasingly important role in transmission because of its rapidly-changing global distribution [2, 3]. With no effective vaccines or effective drugs to prevent or treat this group of diseases, the most effective strategy has been to interrupt the virus transmission cycle by reducing the frequency of contacts between these mosquitoes and the human population [4].

Established vector control methods involving the elimination of larval habitats, the use of physical barriers such as window nets, or those involving insecticides, both larvicides and adulticides, are not sufficient to maintain the populations of these species below the epidemic risk threshold [5, 6]. Moreover, control programs are often hindered by a growing prevalence of insecticide resistance in both of these vectors [7]. The need for complementary vector control tools that are effective, sustainable and environmentally benign, is becoming increasingly clear [8].

The sterile insect technique (SIT) is a species-specific, non-polluting and environmentally benevolent method of insect control. This technique involves the release of massive numbers of artificially-reared sterile male insects that, due to their abundance, outcompete fertile wild males for mating opportunities with wild female insects [9, 10]. The success of SIT-based vector control programs involves an area-wide approach combined with the release of very large numbers of sexually competitive sterile males in urban and peri-urban areas in which high density human populations are at the highest risk of arbovirus transmission [10–12]. Despite important advances in the development of molecular mechanisms for inducing male sterility [13], sterilization by irradiation remains the most practical method to sterilize mosquitoes from a cost and efficiency standpoint.

Large parts of Mexico and Central America are affected by dengue fever and this region recently experienced the rapid invasion of chikungunya in 2014 followed by Zika virus in 2015 [14]. Estimates of the number of symptomatic cases of these diseases greatly exceed the number of confirmed reported cases [15]. In addition, a notable expansion in the geographical distribution of *Ae*. *aegypti* and *Ae*. *albopictus* has been reported that has been attributed to the effects of global climate change and international trade [16–18]. The National Institute for Public Health (INSP) in Mexico is therefore evaluating the possible use of SIT as an additional control measures for *Ae*. *aegypti* and *Ae*. *albopictus*, particularly in the most affected areas of the country, namely coastal areas below 2000 m in altitude. Fortunately, Mexico has a history of successful use of SIT for the control of veterinary pests such as the screwworm [19] and agricultural insect pests such as fruit flies [20]. Consequently, the country is home to one of the world's largest sterile insect production facilities in Chiapas State in southern Mexico [21]. This study aims to generate fundamental results that will be used in the evaluation of the likely efficacy of SIT-based suppression of *Aedes* spp. in southern Mexico in the context of the national technical cooperation project Mex5031 supported by the International Atomic Energy Agency (IAEA).

Previous studies on insect susceptibility to radiation have reported variation across geographical regions [22, 23], and in strains collected at different altitudes [24], the basis for which is likely to be genetic. Given that we had no prior information that would lead us to believe that *Aedes* spp. would not show a genetic basis for variation in sensitivity to irradiation, the objectives of the present study were two-fold: (i) to determine the minimal irradiation dose for full male sterility for *Aedes aegypti* and *Ae*. *albopictus* using local strains and (ii) to determine the effects of irradiation on pupal and adult survival, reproduction and flight ability of both mosquito vector species.

## Materials and methods

### Ethics statement

The present study was performed with the approval of the Ethics in Research and Biosecurity Committees of the Instituto Nacional de Salud Pública (INSP) in Mexico, who reviewed and authorized the procedures described in the project “Aplicación de la técnica del insecto estéril para el control de *Aedes aegypti* y *Ae*. *albopictus* en el sur de Chiapas, México” supervised by J.G.B. Mosquito rearing procedures were performed in the insectary of the Centro Regional de Investigación en Salud Pública (CRISP-INSP). Animal blood was obtained from the municipal slaughterhouse in Tapachula, Chiapas, Mexico where animals are slaughtered in line with local, state and federal guidelines and laws. Mosquito eggs were collected from 12 urban localities in Chiapas, Mexico at sites with unrestricted public access for which specific permission for access or collection was not required. Collection of mosquito eggs did not involve endangered or protected species.

### Laboratory colonies

The laboratory colonies of *Ae*. *aegypti* and *Ae*. *albopictus* used for all experiments originated from eggs collected in 2016 from twelve localities along the Pacific coast of Chiapas, Mexico (S1 File, sheet 1). The twelve populations were subjected to a process of introgression through backcrosses to obtain a genetically diverse strain for each species [25]. Colonies were maintained under controlled conditions at 28 ± 2°C, 80 ± 5% relative humidity (RH), and photoperiod of 14:10 h (light: dark). Larvae were reared at a density of 1.5 larvae/ml in 61x41x7.5 cm plastic trays containing 2000 ml dechlorinated water and were fed with powdered Laboratory Rodent Diet (LabDiet, Fort Worth, Texas, USA), as described previously [25]. Pupae were sexed as a function of body size using a plate separator (John W. Hock, Model 5412, Gainesville, Florida, USA) and the genital lobe was visually checked using a Stemi 508 Stereomicroscope (Carl Zeiss). Adults were placed in 30x30x30 cm acrylic cages with nylon mesh walls (BugDorm 1; Taichung, Taiwan) maintained at 26 ± 2°C, 80 ± 5% relative humidity (RH), and 14 h:10 h (light: dark), photoperiod and supplied *ad libitum* with 10% sucrose solution on a cotton pad. From 4 days post-emergence, bovine blood was provided for three consecutive days using a Hemotek membrane feeding system (PS6B, Hemotek Ltd., Great Harwood, UK).

### Experimental design and pupal irradiation

The irradiator used was a dry storage irradiator (Gamma Beam GB-127, serial number IR-226, Nordion, Otawa, Canada), with a cobalt-60 (^60^Co) source located in the Moscafrut facility in Metapa, Chiapas, Mexico. The dose rate was determined using an ionization chamber RADCAL Model ADDM, USA. Dosages were determined using the Fricke dosimetry system [26] and a Gafchromic film dosimetry system [27]. Male and female pupae were irradiated 24–36 h before adult emergence. The doses tested of 15, 30, 50, 70 and 90 Gy for *Ae*. *aegypti* were obtained from the source with an activity of 14416 Ci over a period of 10 min at distances of 113, 74, 54, 43 and 36 cm from the source, respectively. The doses of 15, 25, 35, 40 and 50 Gy for *Ae*. *albopictus* [28], were obtained over a 10 min period at distances of 113, 80, 67, 62, and 54 cm from the source, respectively. For each replicate, batches of 100 pupae for each dose and sex were placed in 15 ml of dechlorinated water in a 9 cm diameter plastic Petri dish. Three replicates were performed for each dose, species and sex. All subsequent observations on insect survival, flight, reproductive traits and adult survival were performed in the laboratory at 26 ± 2°C, 80 ± 5% relative humidity (RH); 14:10 h light: dark photoperiod.

### Effects of irradiation on pupal mortality

After irradiation, pupae were placed in a 9 cm diameter Petri dish inside an acrylic cage (30x30x30 cm) and left to emerge. At 48 h and 72 h later, dead pupae (that did not respond to the touch of a toothpick) and adults that died during emergence, were counted and removed. All insects that died prior to adult emergence (72 h) were deducted from the total to calculate prevalence of survival for analysis.

### Effects of irradiation on flight ability

To determine the flight ability of adults, 100 pupae of one sex were placed in a 9 cm diameter Petri dish into which a transparent tube, 25 cm in height and 8 cm in diameter, was introduced. This apparatus was placed inside an acrylic cage 30x30x30 cm. Flight ability was measured according to the prevalence of adults that emerged from the pupae and were able to exit the tube over a 48 h period. Pupae that did not emerge were excluded from the results. This procedure was performed three times for all doses, both sexes and both species.

### Effects of irradiation on fecundity and fertility

For each dose, 20 irradiated females and 20 virgin non-irradiated males (both 3 days post-emergence) were selected at random and placed together in cages (30x30x30 cm) with continuous access to 10% sucrose solution. At 4 days post-emergence, females were offered a sheep's blood meal once a day for three consecutive days, using a Hemotek membrane feeding system. After this time each female was placed individually in a 50 ml plastic centrifuge tube containing 10 ml of deionized water and a strip of filter paper (9 x 4 cm) as an oviposition substrate. The tube was sealed with a ventilated mesh lid. The total number of eggs laid by each female (fecundity) and the number of eggs that hatched out of the total number of eggs produced per female (fertility) were determined over a single gonotrophic cycle. To determine the effects of irradiation on males, the experiment was repeated using 20 irradiated males and 20 virgin females that had not been irradiated. Controls consisted of groups of 20 males and 20 females that had not been irradiated. Three replicates (cages) were performed for each treatment.

### Effects of irradiation on ovary length

To examine the effects of irradiation on the size and structure of the ovaries, pairs of ovaries from a group of 15 unfed adult females (7–8 days old) that had been irradiated as pupae were removed and examined. Ovary length was measured as the length of the central longitudinal axis of the ovary [6]. The structure was compared qualitatively. A digital image of each ovary was produced using ZEN 2.3 (blue edition) software for the Stemi 508 Stereomicroscope (Carl Zeiss), fitted with a digital camera. An equal number of ovaries of non-irradiated females of each species were also measured as a control.

### Effects of irradiation on adult survival

For each dose and each species, groups of 20 males and 20 females were placed separately in cages of 13 cm wide x 11.5 cm high x 24.5 cm long. The mosquitoes were provided with continuous access to 10% sucrose solution. Mortality was recorded daily until the death of the last individual. Three replicates (cages) were performed for each dose and species combination.

### Statistical analyses

General Lineal Models (GLM) with a normal error structure were used to compare pupal mortality, fecundity and ovary length. Radiation effects on egg hatch were analyzed using one-way ANOVA and Tukey's post-hoc tests. Radiation treatments that completely eliminated egg production were not included in the analyses because their lack of variance would violate the assumption of homoscedasticity for the analyses. GLM and AVOVA analyses were performed using StatView for Windows, v.5.0 (SAS Institute Inc., USA). Percentage of flight ability values could not be normalized by transformation and were analyzed by Kruskal-Wallis test with mean separation by Dwass-Steel-Critchlow-Fligner pairwise procedure in R using the Jamovi package (www.jamovi.org). The Kaplan-Meier method was used to estimate median survival times of adults held in cages. The Log-Rank test was used to perform pairwise comparisons of survival curves from the different treatments; critical P values were adjusted using the Benjamini–Hochberg false discovery rate procedure to control type I errors in multiple comparisons.

## Results

### Effects of irradiation on pupal mortality

**Fig 1:**
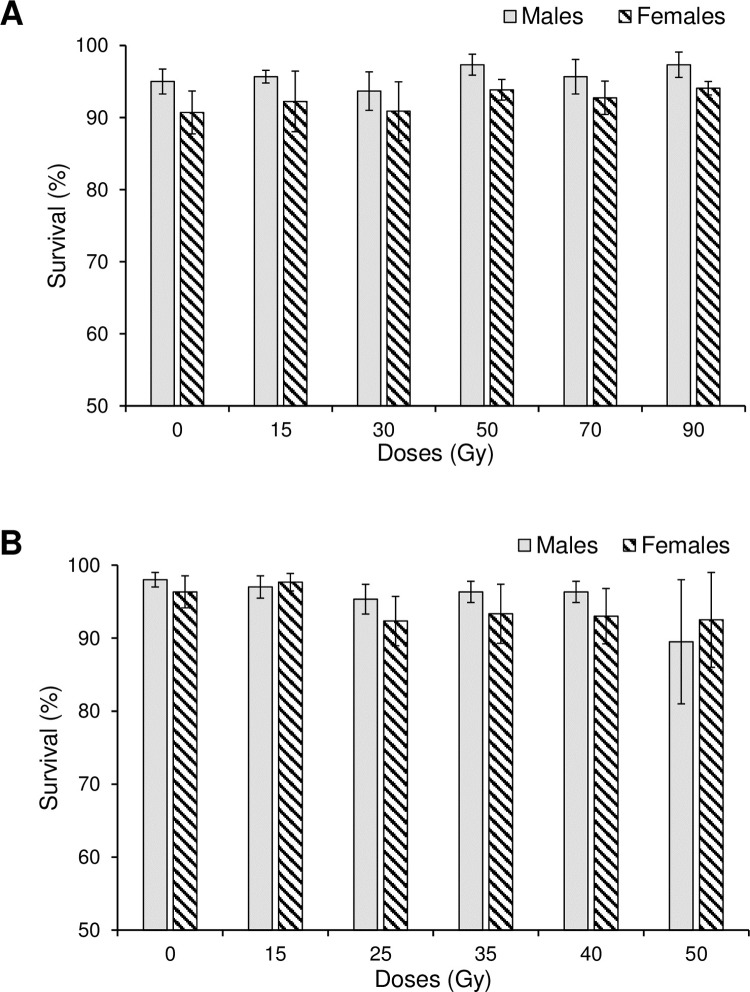
Percentage of survival of males and females following exposure to different irradiation doses. (A) *Aedes aegypti* and (B) *Aedes albopictus*. Vertical bars indicate SE.

The survival of *Ae*. *aegypti* male pupae ranged from 93.6 to 97.3% and did not differ significantly among irradiation treatments, including the control (F_5,12_ = 0.54; P = 0.740) (Fig 1A). The same pattern was observed for female pupae of this species; survival ranged from 90.8 to 94.1% and did not differ significantly for treatments of 15 to 90 Gy or compared to the control (90.7% survival) (F_5,12_ = 0.23; P = 0.938) (Fig 1A).

The survival of *Ae*. *albopictus* male pupae varied between 90.7 and 97.0% among irradiation treatments compared to 98.0% survival in the control (F_5,12_ = 1.07; P = 0.422) (Fig 1B). Survival of *Ae*. *albopictus* female pupae ranged from 90.5 to 97.7% in irradiation treatments compared to 96.3% for the control treatment (F_5,12_ = 0.60; P = 0.703) (Fig 1B).

### Effects of irradiation on flight ability

**Fig 2:**
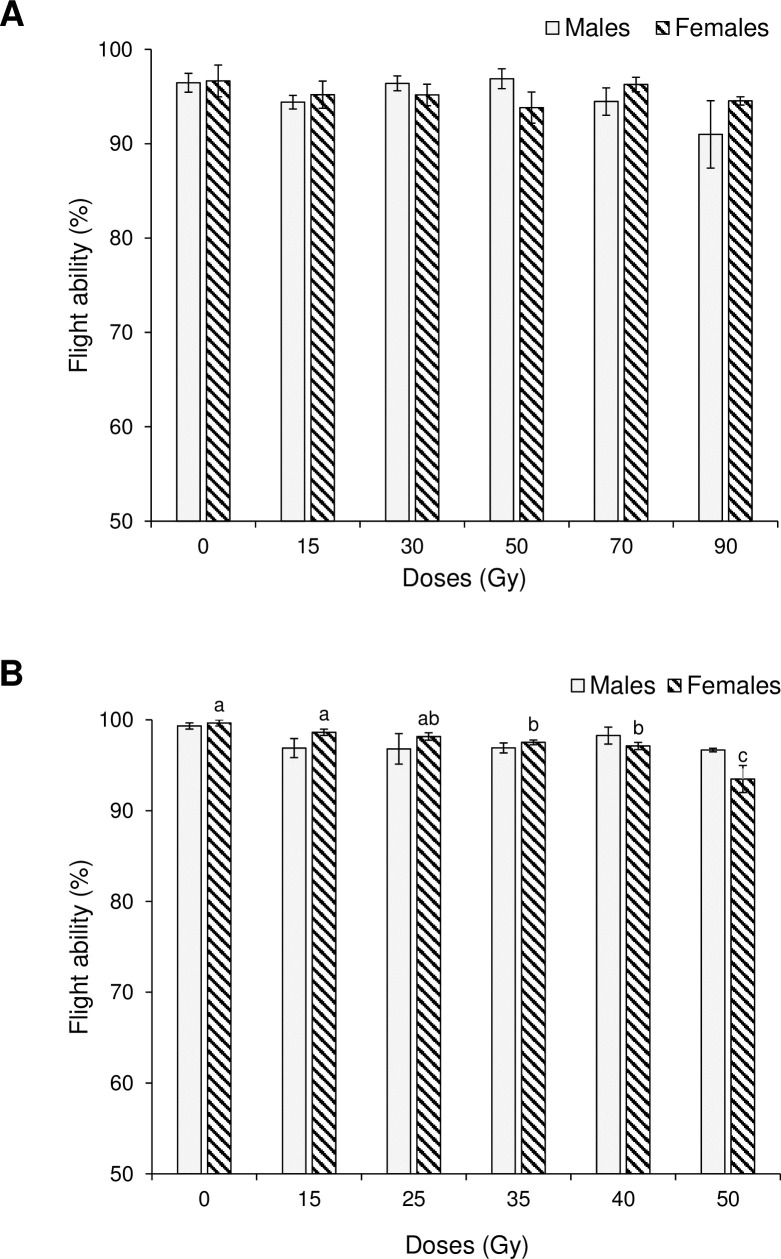
Percentage of flight ability in males and females following exposure to different irradiation doses. (A) *Aedes aegypti* and (B) *Aedes albopictus* Vertical bars indicate SE. Columns without letters did not differ significantly (Kruskal-Wallis, P>0.05). Columns headed by different letters differed significantly for comparisons among treatments applied to females in (B) (Kruskal-Wallis, P≤0.05).

The prevalence of flight ability in adult *Ae*. *aegypti* did not differ significantly among control and irradiation treatments for males (H_5_ = 5.59, P = 0.349), or females (H_5_ = 3.49, P = 0.626), with percentage of flight ability values of 91.0 to 96.5% in males, and 93.8 to 99.7% in females (Fig 2A). Similar results were observed in males of *Ae*. *albopictus* (Fig 2B); flight ability did not differ significantly with irradiation treatments (H_5_ = 7.18, P = 0.207) which ranged from 96.7 to 99.3%. In contrast, significant but small decreases in the prevalence of flight ability were observed in *Ae*. *albopictus* females (H_5_ = 14.07, P = 0.015), which declined from 99.7% in the control, to 97.5–93.5% in the treatments involving doses of 35–50 Gy (Fig 2B).

### Effects of radiation on fecundity

Egg production by irradiated females of *Ae*. *aegypti* over a single gonotrophic cycle was significantly reduced by irradiation at doses of 15 and 30 Gy compared to the non-irradiated control (F_2,151_ = 355.6, P <0.001). The average number of eggs laid per female in all irradiation treatments was lower than that of the corresponding control insects and no eggs were laid by females that had been exposed to doses of 50, 70 or 90 Gy (Table 1). In contrast, the fecundity of non-irradiated control groups did not differ significantly and varied between 97.0–102.3 eggs/female. The percentage reduction in eggs produced by *Ae*. *aegypti* females irradiated at 15, 30 and 50–90 Gy was 24.4%, 99.8% and 100%, respectively.

**Table 1:** Effects of irradiation dose on mean (±SE) egg production of *Ae*. *aegypti* and *Ae*. *albopictus* over a single gonotrophic cycle. Different letters indicate significant differences for comparisons among doses for each species within each column (Tukey, P< 0.05).

Species and dose (Gy)	Irradiated	Non-irradiated
*Ae*. *aegypti*		
0	99.35 ± 4.12^a^	102.33 ± 3.13^a^
15	75.14 ± 3.17^b^	110.33 ± 5.98^a^
30	0.12 ± 0.08^c^	102.62 ± 3.45^a^
50	00.00 ± 0.00*	101.16 ± 3.39^a^
70	00.00 ± 0.00*	100.78 ± 2.81^a^
90	00.00 ± 0.00*	96.97 ± 3.52^a^
*Ae*. *albopictus*		
0	99.46 ± 5.91^a^	91.05 ± 6.07^a^
15	66.81 ± 4.73^b^	89.44 ± 4.82^a^
25	3.28 ± 0.98^c^	92.70 ± 5.01^a^
35	00.00 ± 0.00*	102.49 ± 5.07^a^
40	00.00 ± 0.00*	86.90 ± 5.53^a^
50	00.00 ± 0.00*	102.54 ± 3.54^a^

* Treatments that resulted in a complete loss of egg production were not included in statistical analyses.

Similarly, egg production by *Ae*. *albopictus* was significantly reduced by irradiation at doses of 15 and 25 Gy (F_2,148_ = 150.2, P <0.001) and was completely eliminated in females irradiated at 35, 40 and 50 Gy, whereas control groups of females produced an average of 86.9 to 102.5 eggs/female (Table 1). The reduction in eggs produced by *Ae*. *albopictus* females irradiated at 15, 25 and 35–50 Gy was 32.8%, 96.7% and 100%, respectively.

### Effects of radiation on fertility

**Fig 3:**
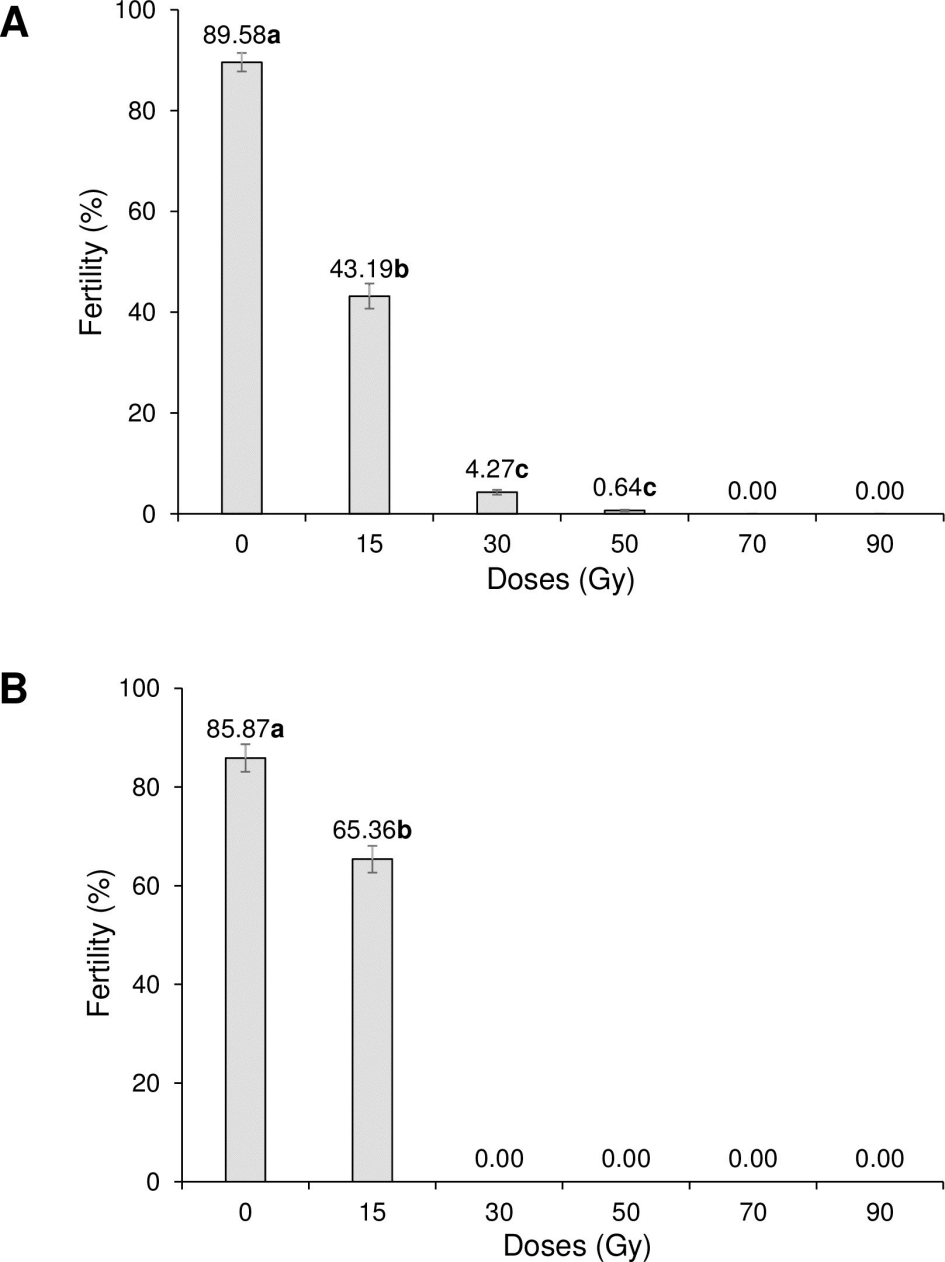
Effects of irradiation on percentage of egg fertility of *Aedes aegypti*. (A) Irradiated males mated with non-irradiated females and (B) irradiated females mated with non-irradiated males. Values above columns indicate percentages. Values followed by identical letters do not differ significantly (Tukey, P>0.05). Vertical bars indicate SE.

In irradiated *Ae*. *aegypti* males that mated with non-irradiated females, fertility was reduced from 89.58% in the control to close to zero in the 50 Gy treatment (F_3,171_ = 825.2, P < 0.001) and was completely eliminated following irradiation at 70 and 90 Gy (Fig 3A). In irradiated *Ae*. *aegypti* females that mated with fertile males, control fertility was 85.87% and was reduced to 65.36% at 15 Gy (F_1,93_ = 27.0, P <0.001) and to 0% following irradiation at 30 to 90 Gy (Fig 3B).

**Fig 4:**
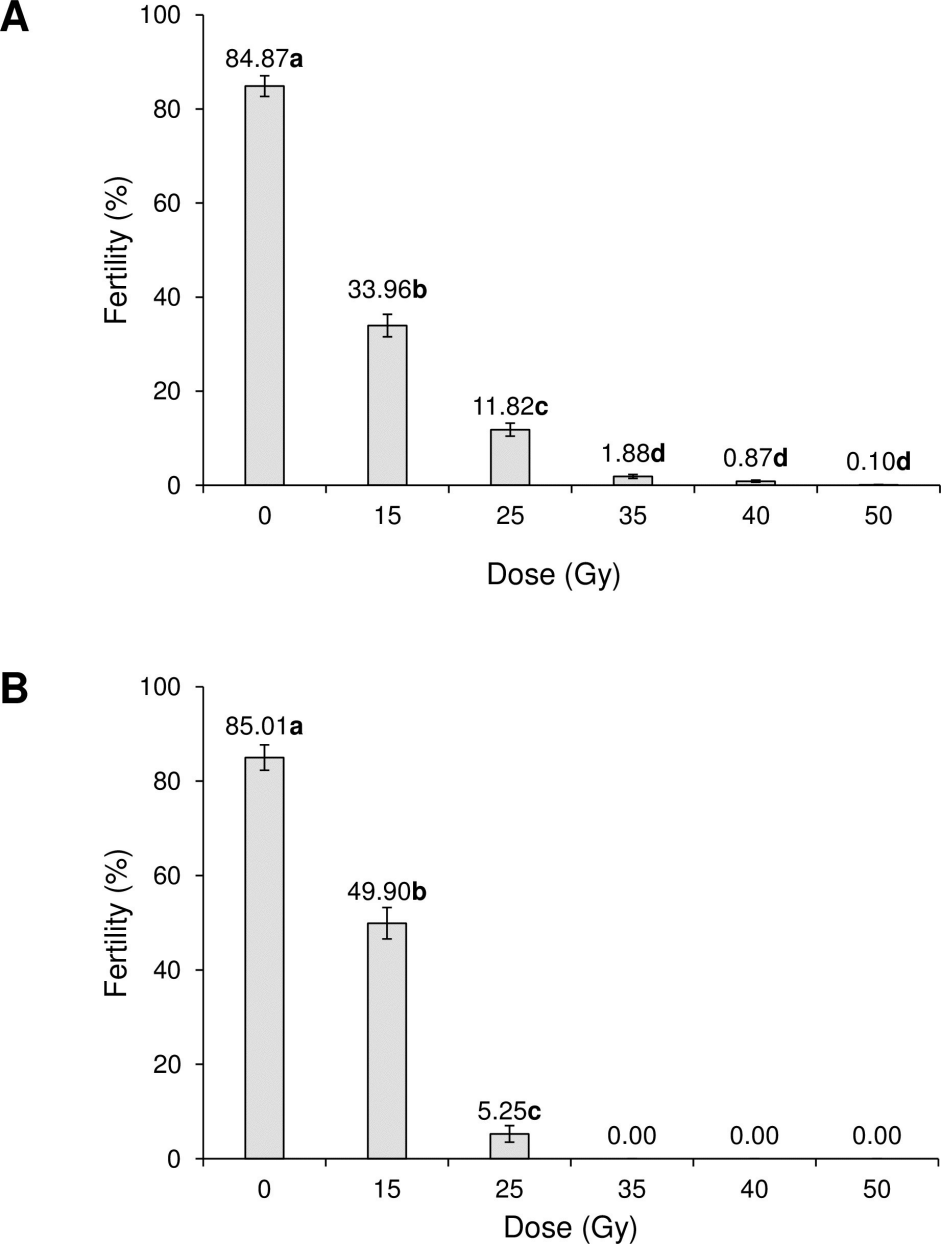
Effects of irradiation on percentage of egg fertility of *Aedes albopictus*. (A) Irradiated males mated with non- irradiated females and (B) irradiated females mated with non-irradiated males. Values above columns indicate percentages. Values followed by identical letters do not differ significantly (Tukey, P>0.05). Vertical bars indicate SE.

In the case of *Ae*. *albopictus*, for irradiated males that mated with non-irradiated females, increasing doses of radiation resulted in a steady reduction in fertility from 84.87% in control insects to close to zero (0.10%) in males exposed to the highest dose of 50 Gy (F_5,288_ = 534.0, P <0.001) (Fig 4A). In contrast, in females that mated with non-irradiated males, fertility was 85.01% in control insects and fell significantly in the 15 and 25 Gy treatments (F_2,148_ = 249.9, P <0.001), and was completely eliminated in treatments involving 35–50 Gy (Fig 4B).

### Effects of irradiation on ovary length

**Fig 5:**
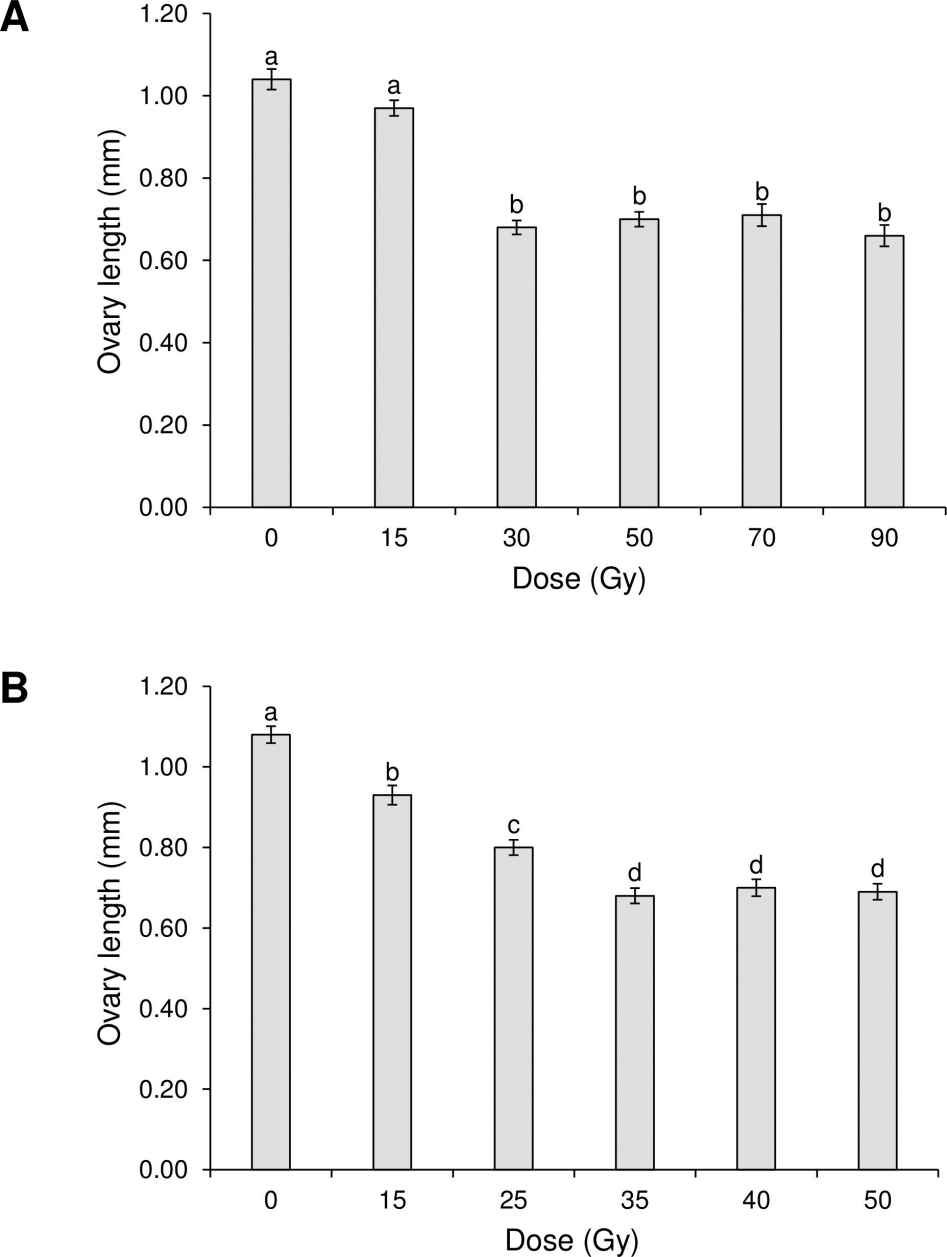
Effect of irradiation on mean ovary length. (A) *Aedes aegypti* (A) and (B) *Aedes albopictus*. Columns headed by identical letters do not differ significantly (Tukey, P>0.05). Vertical bars indicate SE.

The mean ovary length of *Ae*. *aegypti* was not significantly affected by exposure to 15 Gy (Fig 5A), but was significantly shorter in females that had been exposed to 30 to 90 Gy, compared to control insects (F_5,168_ = 52.0, P <0.001). Irradiation also resulted in reduced ovary length in *Ae*. *albopictus* compared to control insects at all doses and were shortest in females that had been exposed to 35 to 50 Gy (F_5,240_ = 60.7; P <0.001) (Fig 5B). Microscopic examination revealed that fewer follicles were present in the ovaries of irradiated females, although these were not quantified.

### Effects of irradiation on adult survival

**Fig 6:**
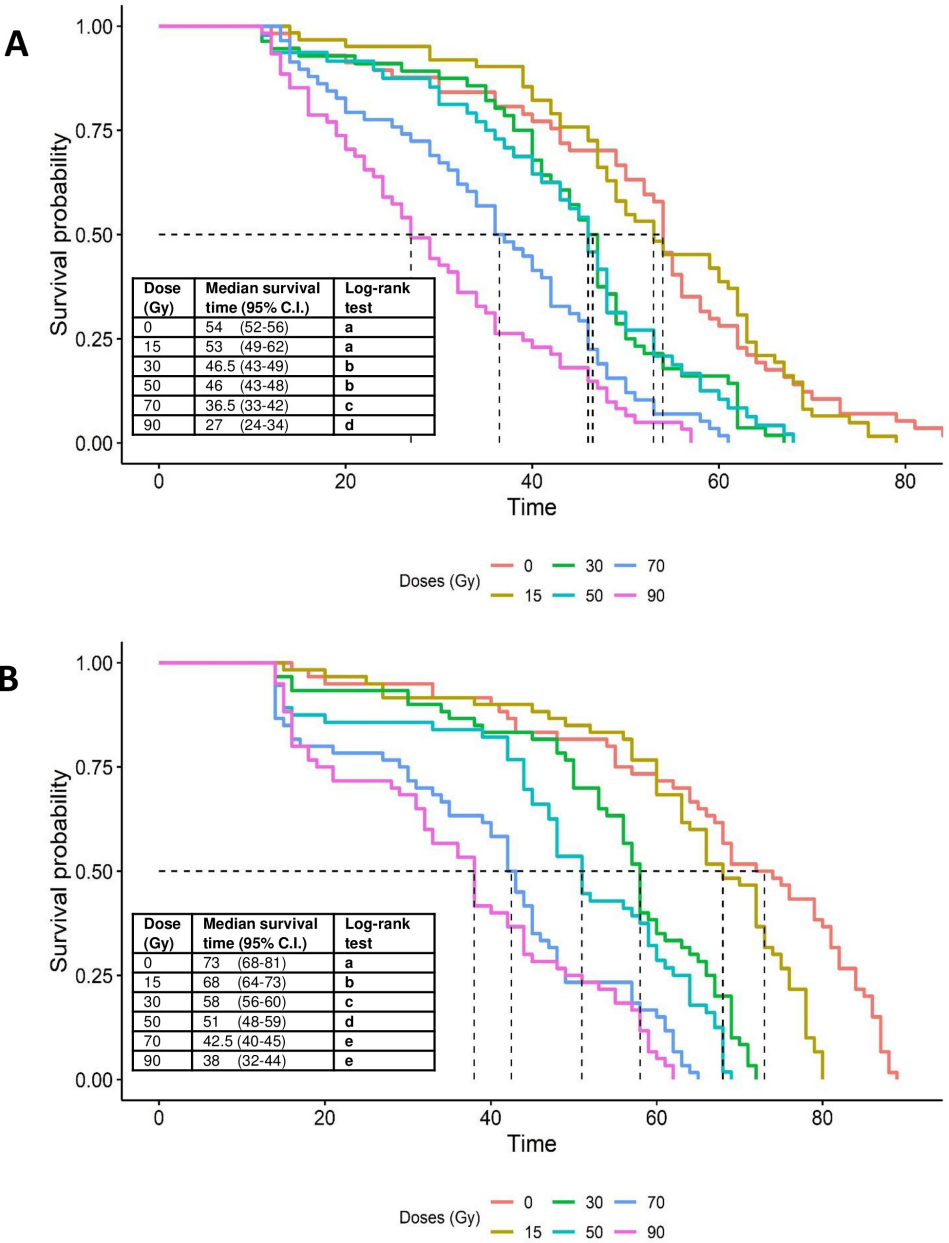
**Kaplan-Meier survival curves for *Ae*. *aegypti* adults (A) males and (B) females following exposure to different doses of radiation in the pupal stage.** Pairwise comparisons of survival curves was performed by log-rank test with Benjamini–Hochberg false discovery rate adjusted P-values. Dashed lines indicate median survival times for each radiation treatment.

The survival of both sexes of *Ae*. *aegypti* was significantly reduced by irradiation (Fig 6A and 6B). Survival of males was significantly reduced at doses of 30 Gy or more compared to the control (log-rank test, P <0.001). Accordingly, median survival time decreased from 54 days for control males to 27 days for males exposed to 90 Gy (Fig 6A). For females of *Ae*. *aegypti*, survival was significantly reduced in all radiation treatments compared to the control (log-rank test, P <0.001) and median survival time decreased steadily from 73 days in control to 38 days in the 90 Gy treatment (Fig 6B).

**Fig 7:**
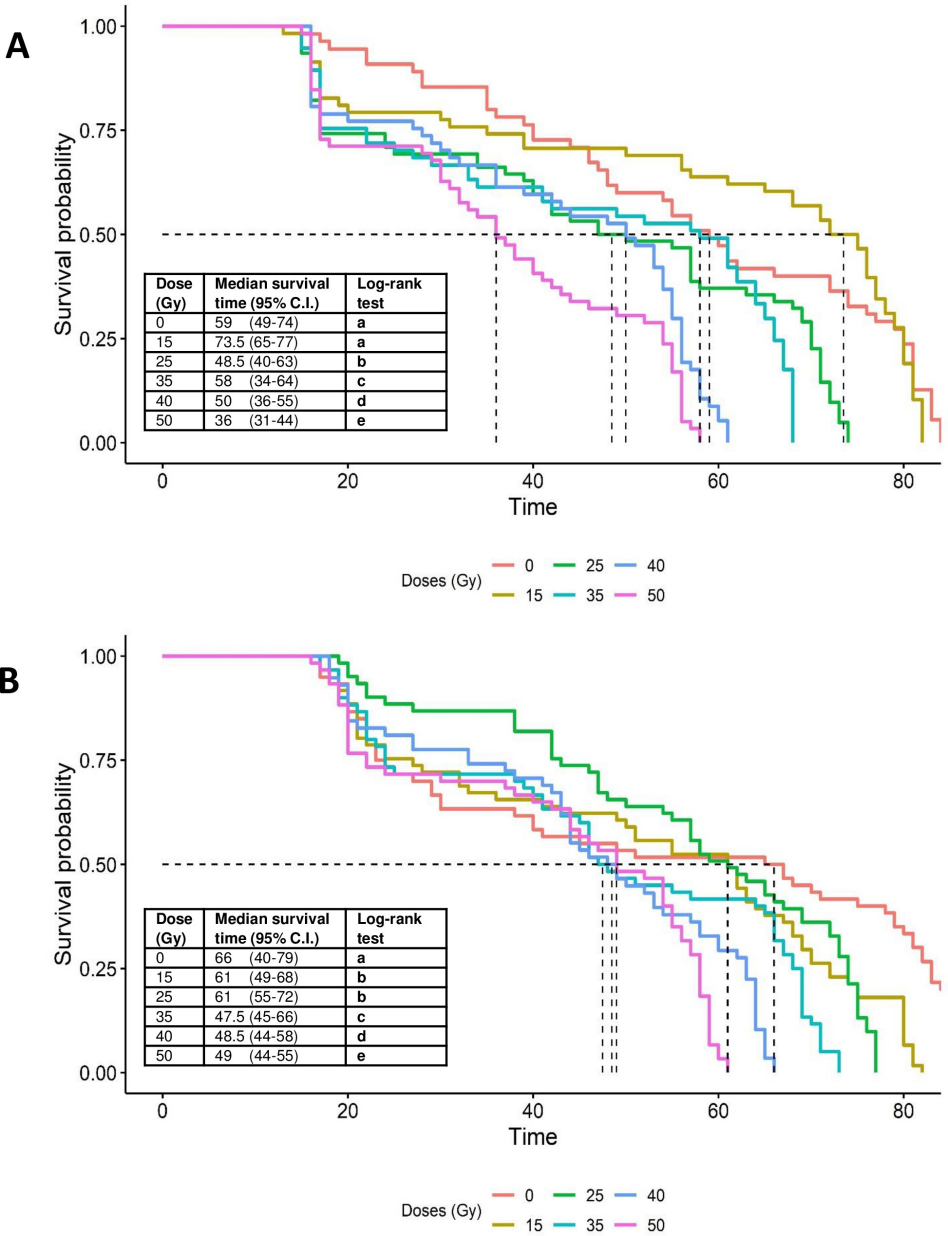
**Kaplan-Meier survival curves for *Ae*. *albopictus* adults (A) males and (B) females following exposure to different doses of radiation in the pupal stage.** Pairwise comparisons of survival curves was performed by log-rank test with Benjamini–Hochberg false discovery rate adjusted P-values. Dashed lines indicate median survival times for each radiation treatment.

In the case of *Ae*. *albopictus*, adult survival time was also significantly affected by irradiation in males and females (Fig 7A and 7B). In males, survival was significantly reduced at radiation doses of 25 Gy or more compared to the control (log-rank test, P <0.001). Median survival time in males was higher in the 15 Gy treatment (73.5 days) compared to the control (59 days), but this difference was not significant (log-rank test, P = 0.645) and in general, median survival time decreased with increasing radiation dose (Fig 7A). In females, survival was reduced significantly in all radiation treatments compared to the control (log-rank test, P <0.001). Median survival time in females decreased from 61 days in the 15 and 25 Gy doses to 49 days in the 50 Gy dose, compared to 66 days in control insects (Fig 7B).

## Discussion

The effect of irradiation on immature survival, flight capacity, reproductive characteristics and adult survival were compared for *Ae*. *aegypti* and *Ae*. *albopictus* across a range of doses. In general, higher doses had adverse effects on reproductive traits in both species and fertility was eliminated at doses of 30 to 70 Gy, depending on sex and species.

In the present study, radiation at any dose did not significantly influence the survival of pupae of *Ae*. *aegypti* and *Ae*. *albopictus* up to the point of adult emergence. In previous studies, increased mortality was not observed in *Ae*. *albopictus* exposed to doses up to 40 Gy from an X-ray tube source [6], or in male pupae of *Ae*. *albopictus* exposed to X-rays at doses up to 60 Gy [29]. Similarly, pupal mortality was not adversely affected in *Anopheles arabiensis* at doses as high as 100 Gy [30], whereas pupal viability, survival and adult emergence decreased with increasing dose between 100 and 1000 Gy when *Ae*. *aegypti* were treated in the pupal stage [31].

The selection of radiation dose used to induce sterility represents a trade-off between effective sterilization and male competitiveness [32]. The prevalence of sterility in groups of treated insects increases with dose, as does the magnitude of adverse effects on male quality and mating competitiveness [29]. An appropriate decision on treatment dose therefore requires quantitative information on the influence of dose and other process-related issues (insect stage, age, physical conditions, etc.) on sterility and indicators of insect quality, such as those examined in the present study.

The flight ability of both sexes of *Ae*. *aegypti* and males of *Ae*. *albopictus* was not significantly compromised following irradiation at any of the doses tested, whereas females of *Ae*. *albopictus* were adversely affected at doses of 50–90 Gy. The design of the device used to evaluate flight ability was based on an 8 cm diameter x 25 cm tall tube previously used for quality control of irradiated fruit flies [33]. Recently, a different device for testing mosquito flight ability was described, comprising a bundle of narrow (0.8 cm diameter) acrylic tubes placed above a holding chamber and with fan-assisted dispersal of a volatile lure placed above the tubes [34]. These authors reported significant decreases in the prevalence of escape from the device in adult males of *Ae*. *aegypti* and *Ae*. *albopictus* that had experienced doses of 90 and 40 Gy, respectively. These findings suggest that the device used in the present study was less effective at detecting dose-dependent flight effects than the device developed by Culbert et al. [34].

Clearly flight is the primary mechanism for dispersal and is essential for vector activity [35]. Both irradiated and control males of *Ae*. *albopictus* have two diurnal peaks in flight activity in the morning and late afternoon [36]. In another study, irradiated males that had been sterilized by exposure to 35 Gy were more active and flew faster and further than control insects, although this behavior was modulated by nutritional conditions [35]. Positive biological effects at low doses of radiation may be indicative of hormesis [37], a phenomenon reported in the Caribbean fruit fly, *Anastrepha suspensa* [38].

For both *Ae*. *aegypti* and *Ae*. *albopictus*, egg production of females that mated with irradiated males was similar for all the treatments compared to the control. Indeed, in other studies, *Ae*. *aegypti* females that mated with sterile males continued to lay eggs even when males have been exposed to doses in the range 10 to 300 Gy [39, 40]. Similarly, *An*. *arabiensis* females that mated with irradiated males that had been exposed to 25 to 100 Gy was similar for all treatments and insemination studies indicated that irradiation did not affect the males' ability to impregnate fertile females under laboratory conditions [30].

When irradiation treatments were applied to females, doses up to 25 Gy did not affect the fecundity of *Ae*. *aegypti*, whereas doses of 30 to 50 Gy resulted in a significant reduction in egg production in treated females [40]. Causes for the lack of fecundity may include damage to the ovarian tissue resulting in the inability to produce eggs, or the inability to mate [40, 41].

The effects of irradiation on egg fertility varied with species, dose and sex. In *Ae*. *aegypti*, the fertility of eggs fathered by irradiated males that mated with non-irradiated females was eliminated at doses of 70 and 90 Gy, whereas the eggs of irradiated mothers that had mated with fertile males completely lost fertility at doses of ≥30 Gy, indicating that females were more susceptible to irradiation than males. Similar findings were reported by Shetty et al. [40], who observed decreased egg fertility following male exposure to doses of 20–50 Gy in *Ae*. *aegypti*. The reduced fertility was transmitted to the following three generations indicating an inherited change to the germ line [40].

In *Ae*. *albopictus*, the egg fertility produced by irradiated males of that mated with non-treated females was reduced to less than 1% by doses of 40 and 50 Gy. Previous studies showed that the fertility of eggs fathered by irradiated *Ae*. *albopictus* males was eliminated at doses of 40 to 80 Gy [28], and was reduced to 7 and 4% at doses of 35 and 40 Gy, respectively [42].

In general, females of both *Ae*. *aegypti* and *Ae*. *albopictus* were more susceptible to irradiation than males, with a complete loss of egg fertility at doses of 30 Gy or above. Indeed, a clear negative correlation between egg fertility and irradiation dose has been reported across several species of mosquitoes [6, 28, 40, 42].

The sterilization process is important in determining the quality of the released male insects and their ability to compete with the wild population [43]. In our study, 0.1% fertility in males was obtained following treatment of *Ae*. *albopictus* at a dose of 50 Gy, which compares to previous studies in which 1% fertility was observed at doses in the range 30–35 Gy [28] or 7 and 4% fertility at doses of 35 and 40 Gy, respectively [12]. In the present study, *Ae*. *aegypti* were fully sterilized at 70 Gy, in line with the findings of Hallinan and Rai [44], whereas Weidhaas and Schmidt [45] obtained 99.9% sterility at 78 Gy.

The length of the ovaries of irradiated females of both species decreased in the present study and suffered gross damage as irradiation dose increased (Fig 5A and 5B and S1 File). Reduced egg production and loss of fertility have been attributed to somatic and germ-line cellular damage in the ovaries of *Ae*. *abopictus* [6], whereas dose-dependent reductions in the size of testes and ovaries and reduced oocyte reabsorption were reported following irradiation of the lepidopteran pest, *Plodia interpunctella* [46].

Adult survival in laboratory cages was reduced by irradiation in both *Ae*. *aegypti* and *Ae*. *albopictus*. For the sterilizing doses identified in the present study (35 Gy for *Ae*. *albopictus* and 50 Gy for *Ae*. *aegypti*), median survival time was reduced by just 15% in *Ae*. *aegypti* males irradiated at 50 Gy with respect to the control (Fig 6A), whereas for *Ae*. *albopictus* males irradiated at 35 Gy, median survival time was almost identical to that of the control (Fig 7A). Higher doses of radiation were detrimental to adult longevity in both species. Reduced longevity following exposure to high doses of radiation have been reported previously in *Ae*. *aegypti* [47, 48] and *Ae*. *albopictus* [49, 50]. Similar effects have also been observed in anopheline species at doses beyond 80 Gy [30, 40]. Although irradiation is intended to generate dominant lethal mutations in tissues that have high rates of cell division (germ cells), the process is non-specific and can also damage somatic cells, which, in combination with an increase in oxidative stress, can result in cellular death [10, 30]. Indeed, one of the most common effects of somatic damage is reduced longevity [30].

The impact of irradiation on adult longevity appears to be higher during the early stages (16–24 h) of pupal development compared to the later stages (24–48 h) in *Ae*. *albopictus* [28]. In the present study male pupae were irradiated at 24–36 h before adult emergence and effects on survival times were species specific and dose dependent (Fig 6A and 6B). Interestingly, radioprotective compounds such as dilute ethanol, beer and trimethylglycine have been shown to be effective in reducing somatic damage and increasing longevity in adult males that had received a sterilizing dose of X-rays in the adult stage [51].

Following the release of sterile males, the duration of adult survival is likely to influence the number of mating opportunities and the area over which sterile males disperse. Of course, in natural habitats, the survival of sterile male insects is much lower than under laboratory conditions, where insects have ready access to food and are protected from predators, pathogens and adverse climatic conditions [52, 53]. In fact, the relatively small reductions in adult survival times that we observed in irradiated laboratory-reared mosquitoes are unlikely to have a large influence on average survival time in nature, which typically has been estimated in the range 2–11 days in *Ae*. *aegypti* [54–58], and approximately 11 days in *Ae*. *albopictus* (value estimated from figure in [58]), with males having a shorter average life expectancy under natural conditions than females [55, 59].

In conclusion, irradiation of *Ae*. *aegypti* males at a dose of 50 Gy resulted in 99% sterility with little adverse effect on adult survival time. In the case of *Ae*. *albopictus*, the dose of 35 Gy resulted in 100% sterility, in line with previous studies [25, 32, 39], and little reduction in adult survival time. Consequently, based on the results of this study and the impact of radiation on the fertility, flight ability and lifespan of irradiated males, we recommend doses of 35 Gy and 50 Gy for sterilization of *Ae*. *albopictus* and *Ae*. *aegypti*, respectively. The results obtained in this study will be applied at the field cage and pilot study scales during a stepwise evaluation of the efficacy of SIT-based suppression of *Ae*. *aegypti* populations in southern Mexico within the IAEA-funded project MEX5031.

## Supporting information

S1 File Original data from all studies. (XLSX)
